# Mechanisms of Action of Ig Preparations: Immunomodulatory and Anti-Inflammatory Effects

**DOI:** 10.3389/fimmu.2014.00690

**Published:** 2015-01-12

**Authors:** Andrea Matucci, Enrico Maggi, Alessandra Vultaggio

**Affiliations:** ^1^Immunoallergology Unit, Department of Biomedicine, Policlinico di Careggi, Florence, Italy; ^2^Immunology and Cellular Therapies Unit, Department of Biomedicine, Centre Denothe, Policlinico di Careggi, University of Florence, Florence, Italy

**Keywords:** immunoglobulin, immunodeficiency, immunoregulation of Ig, Ig replacement therapy, antibody defect

## Abstract

Primary immunodeficiency (PID) disorders that predispose patients to recurrent infections require immunoglobulin (Ig) replacement therapy. Ig replacement therapy has been stated as beneficial, although the optimal IgG trough level to be maintained over time in order to minimize infectious risk has not been established. The most common route of administration of Ig has been intravenously, although there are different options, one of them being the subcutaneous route. Ig replacement therapy has been a life-saving treatment for patients suffering from primary and secondary antibody immunodeficiency. The key role of regular Ig replacement in patients with antibody deficiencies is related to the ability to provide specific antibodies that could not be produced by these patients as demonstrated by the reduction of severe infections such as meningitis and pneumonia. The therapeutic benefits of Ig may also be due to an active role in various anti-inflammatory and immunomodulatory activities, which may complicate the clinical picture of PID. Anti-inflammatory activities are seen more generally when intravenous Ig is administered at high dose. The immunomodulatory and anti-inflammatory activities are important not only in the treatment of autoimmune diseases but also in patients suffering from immunodeficiency.

## Introduction

Primary immunodeficiency (PID) disorders that predispose patients to recurrent respiratory, skin, and gastrointestinal infections, require immunoglobulin (Ig) replacement therapy. Common variable immunodeficiency disease (CVID) is one of the most frequent PID characterized by decreased serum levels of all Ig isotypes and recurrent bacterial infections encompassing a heterogeneous group of diseases whose unifying feature is hypogammaglobulinemia (1). The etiopathogenesis of CVID has not been yet fully elucidated. Major progress toward elucidating CVID has been achieved with the identification of defects not only in B-cells, which are directly responsible for antibody production, but also in other immune cells implicated in the generation of an effective humoral response, including antigen-presenting cells (APC) and, in a significant proportion of these patients, helper T cells (2–4). Overall, the disorder is characterized by a defective antibody production by B lymphocytes. Although the number of B-cells may be normal, patients with CVID show extremely decreased serum IgG, IgA, and, occasionally, IgM concentrations. The treatment of choice for CVID patients is replacement Ig therapy. A common route of administration of Ig has been intravenously (IVIg) although today there are different options, one of them being the subcutaneous route (SCIg). Ig replacement therapy has been a life-saving treatment for patients suffering from primary and secondary antibody immunodeficiencies, in fact, a recent published meta analysis of clinical trials in PID quantitatively confirms that trough IgG levels directly impact clinical outcomes (5). However, the optimal IgG trough level to be maintained over time in order to minimize infectious risk, has not been established and probably it should be individualized (6–8). The key role of regular Ig replacement in patients with antibody deficiencies is related to the ability to provide specific antibodies that could not be produced by these patients as demonstrated by the reduction of severe infections such as meningitis and pneumonia (9). The therapeutic benefits of Ig may also be due to an active role in various anti-inflammatory and immunomodulatory activities. In fact, clinical and immunopathological aspects of the association between CVID and autoimmune or inflammatory disorders have been extensively reported in a number of patients (10, 11). On the other hands, Ig preparations other than antibodies to superantigens and pathogens also contain numerous soluble proteins with biologic activity such as cytokines, chemokines, soluble cytokine receptors, and receptor antagonists. In fact, since they were first administered to patients with antibody deficiency disorders over 50 years ago, human intravenous Ig preparations have been used successfully to treat a rapidly increasing number of autoimmune and inflammatory disorders, among which are a series of cutaneous autoimmune and inflammatory diseases (12). Despite the identification of protective, immunomodulatory, and anti-inflammatory activities in various diseases, the benefits of Ig are not easily explained and probably depend by several mechanisms. Anti-inflammatory activities are seen more generally when intravenous Ig is administered at high dose. The immunomodulatory and anti-inflammatory activities are important not only in the treatment of autoimmune diseases but also in patients suffering from immunodeficiency. In this article, the protective and immunoregulatory mechanisms are summarized.

## Immunoglobulin Preparation

The major component of IVIg and SCIg preparations is the IgG fraction, which is pooled from human plasma of several thousands of donors using a procedure that varies somewhat between manufacturers, but results in a product that is a relatively pure concentrate of intact monomeric IgG, with a half-life of 3 weeks, and with small amounts of IgA and IgM. IgG subclasses (IgG1, IgG2, IgG3, and IgG4) in IVIg and SCIg products have a distribution similar to that found in normal human plasma. IgG aggregates are virtually absent from the majority of Ig preparations even if up to 1–10% of IgG can be found in dimeric form in most of IVIg preparations. The processes of purification have the potential to adversely affect the final quality and biological activity of IVIG/SCIG in terms of efficacy and safety (13). Natural antibodies and autoantibodies are prominent in commercial preparations. A wide range of specificities have been identified within Ig preparations including idiotypes of Ig itself, T cell receptor, cell surface molecules such as CD4, CD5, Fas, BAFF, cytokines, and cytokine receptors, such as IL-1; IL-6, tumor necrosis factor (TNF)-α, chemokine receptors, molecules such as sialic acid binding Ig-like lectin (Siglec)-8 and -9 or major histocompatibility complex (MHC) molecules; natural autoantibodies of IgG isotype directed against the human FcγRIII (CD16) and FcγRII (CD32) (14–16).

## Mechanisms of IVIg in the Correction of Humoral Defects

**Figure 1:**
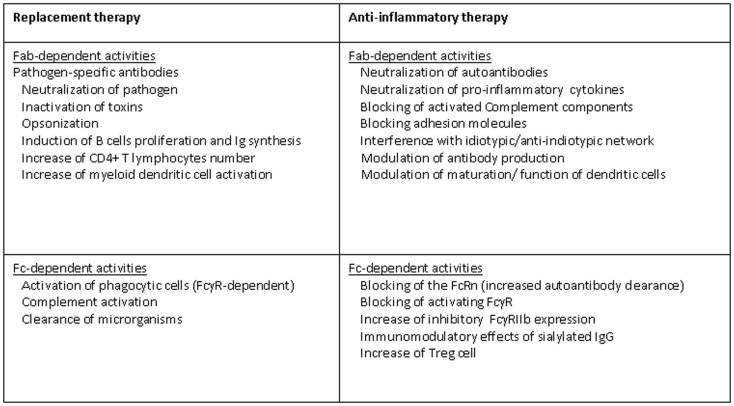
**Mechanisms of action of IVIg in PID**.

The underlying mechanisms of therapeutic effects of IVIg/SCIg in PID are not completely understood, the major aim is to prevent life-threatening bacterial or viral infections (Figure 1). IVIg/SCIg act mainly as a reconstitution therapy, providing patients with pathogen-specific antibodies able to protect from infectious. The unique structure of the Ig molecule ensures the large repertoire of specificities of the antibodies. To maintain the polyclonal nature of the antibody repertoire that is normally present in serum of healthy subjects naturally exposed to microbial agents or submitted to vaccines is a crucial step during the preparation of commercial Ig. Considering that IVIg preparations are generated from adults who have been vaccinated and have encountered a multitude of pathogenic microorganisms, serum IgG can comprise more than 100 million unique specificities. The clinical and functional activities can be distinguished by the infused amounts. In fact, a monthly Ig dosage of at least 400 mg/kg body weight is recommended and is most often sufficient as replacement therapy (17). The activities of Ig molecules present in IVIg/SCIg preparations, such as bactericidal effect through complement system activation, viral neutralization, inactivation of toxins, and opsonization, are crucial for the induction of an effective immune response against several microorganisms and their toxic products. As known, IgG antibodies include two functional portions as the F(ab′) fragment, which is responsible for antigen recognition, and the fragment crystallizable (Fc), crucial for activating the mechanisms of immunity by interacting with Fcγ receptors on B-cells and other cells of the innate immune system belonging to the phagocytic system. The Fc fragment is also crucial for the activation of complement and for the clearance of microorganisms (18). B-cells from several CVID patients seem to be not intrinsically defective as they express CD40 and proliferate significantly upon anti-CD40 stimulation. Interestingly, IVIg at “replacement dose” (10 mg/ml) are able to interact with B-cells, also inducing significantly higher proliferation of B-cells than anti-B cell receptor (BCR) stimulation alone. In addition, IVIg replacement is able to induce Ig synthesis *ex novo* by B-cells (19). Therefore, IVIg therapy, at least in some CVID, is able to modulate B cell functions and it is a passive transfer of antibodies. As previously mentioned, PID are a heterogeneous group of disorders that affect distinct components of the innate and adaptive immune system. Defects not only in B-cells, which are directly responsible for antibody production, but also in other immune cells such as APC and T helper (Th) cells, represent the molecular basis of CVID (20, 21). It has been shown that in CVID patients the humoral defects may be associated with immunological abnormalities of T cell compartment and myeloid dendritic cells (mDC), characterized by low counts of CD4+ T cells, high expression of HLA-DR and CD38 (also on CD8+ T cells), suppressed number of mDC, highly positive for CD80 and CD83. Several of these cellular perturbations are partially corrected by the treatment with IVIg. In fact, the introduction of therapy may lead to CD4+ T cell recovery and decline in CD8+ T cells and mDC activation. These effects are likely sustained by an improved immune control of infections due to humoral reconstitution (22).

## Mechanisms of Action of IVIg in Comorbidity of PID

The use of IVIg has been firmly established for the treatment of a wide variety of autoimmune and inflammatory diseases, due to their immune-regulatory and anti-inflammatory effects (Figure 1). Some of these autoimmune diseases may be a comorbidity of PID, especially CVID, thus sustaining an additional role, beyond the antibody replacement, for IVIg in the treatment of immunodeficiencies. For example, the immunoregulatory functions of IVIg in PID patients explain the therapeutic effects showed in autoimmune hemolytic anemia and/or immunothrobocitopenia, probably by blocking the clearance of opsonized target cells or by suppressing antibody-dependent cell-mediated cytotoxicity. This potential was first revealed when IVIg, used to treat a patient with antibody deficiency, were able to restore platelets count when concomitant thrombocytopenia occurred (23). The way in which IVIg exert their immunomodulatory effects remain unclear, with many pathways, probably mutually non-exclusive, in the innate and adaptive immune systems being potentially targeted. At least a percentage of immune modulatory effects of IVIg are dependent upon the interaction between the Fc portion with the Fcγ receptors expressed on the surface of cells as macrophages, B-cells, natural killer (NK) cells, plasma cells, and platelets (18). For example, as previously mentioned, it has been clearly demonstrated that Fc fragment of IgG can be sufficient to ameliorate immune-mediated thrombocytopenia in humans (24), by suppressing the phagocytosis of platelets via an Fc-dependent mechanism instead of preventing autoantibodies from binding to cells (25). Studies performed both in mice and humans confirmed that IVIg infusion is able to inhibit the mononuclear phagocytic system, usually activated by immune complexes through activating of low-affinity FcγRs (26). However, there is no direct proof that IVIg block the binding of immune complexes to FcγRs. The Fc portion of Ig not only impacts the function of activating Fc receptors but also increase the expression of inhibitory FcγRIIB on macrophages (27). Recent studies in animal models of idiopathic thrombocytopenic purpura suggest that IVIg, increasing the expression of the Fcγ receptor IIB, may reset the threshold for cell activation by immune complexes (18, 25). In other words, IVIg should be able to shift the FcγR-dependent balance of activating and inhibitory signals even more toward cell inhibition of innate immune effector cells.

A mechanism implicated in immune-regulatory function of IVIg preparation is also the effect on the balance between pro- and anti-inflammatory cytokines. To this effect, antibodies to IL-1 and TNF-α have been identified in addition to a down-regulation of such cytokines (28). Furthermore, IVIg induce anti-inflammatory cytokines such as IL-10, TGF-β, and IL-1ra from monocytes/macrophages (28, 29). In our hands, in IVIg-treated PID patients the raising of IL-10 after administration of therapy was not observed in those with associated granulomatous lung disease, thus suggesting the lack of the induction of regulatory cytokines in such subgroup of patients. Dendritic cells represent an important source of pro-inflammatory and anti-inflammatory cytokines and a modulation of cytokine secretion has been shown, characterized by a decrease of IL-12 production and enhanced secretion of IL-10 has been described. DC maturation, activation, and survival are also targeted by IVIg, thus affecting the overall APC activity with subsequent inhibition of adaptive T cell response, including autoreactive cells (30). This latter effect is of potential relevance considering the beneficial action in autoimmune conditions.

Dendritic cells are professional APC with superior capacity to present both MHC-restricted and CD1-restricted antigens. DCs may adapt their CD1 antigen presentation machinery according to signals in the microenvironment. A role for IgG in regulating the expression of CD1 molecules in human DCs has been shown in *in vitro* experiments. In particular, it has been found that the level of exposure to IgG regulates the CD1 expression profile during DC differentiation, and that this is mediated by FcγRIIa. This in turn determines whether the DCs will be biased toward activation of CD1d-restricted regulatory NKT cells or T cells specific for lipid antigens presented by CD1a, CD1b, and CD1c (31). Furthermore, results obtained from patients with CVID indicate that mDCs express elevated levels of CD1a and CD1b in the presence of low levels of IgG *in vivo*, and that this aberrant expression pattern is normalized after IVIg therapy (32). These findings are important for our understanding of diseases associated with Ig deficiencies and their treatment with IVIg.

Concerning the IVIg effects on apoptosis, triggered by the interactions between CD95 (Fas) and its ligand (CD95L, FasL), controversial data are present in literature and probably both agonist and antagonist properties may be attributed to IVIg. In CVID, an increased expression of Fas on CD3+ T cells has been demonstrated and a further increase was shown after IVIg therapy (33). In the context of autoimmune disease as comorbidity of PID, the process could be important in that specific harmful T cells may be deleted through apoptosis.

The complement system is a first line of defense against invading microorganisms, but if its activation occurs under inappropriate circumstances, this system may be not beneficial leading to complement-mediated disorders characterized by cell lysis and tissue damage. Ig plays an intriguing role in complement and regulation. IVIg preparation contain pathogen-specific antibodies and autoantibodies able to activate the effector system, but on the other hand, the rest of the circulating Ig pool (mainly of IgG and IgM isotype) have the capacity to attenuate damaging effects of activated complement fragments. This latter effect seems to be related to a scavenger action toward complement fragments that is dependent on a low-affinity interaction between the fragment in question and various Ig regions (Fc binding to C3b/C4b; Fab binding to C3a/C5a). Such immunocomplexes are then removed by the reticuloendothelial system (34).

Even if many immunoregulatory effects have been ascribed to IVIg, it remains unclear why high doses of IVIg are required to obtain this activity. Some insights were gained when the role of IgG glycosylation was addressed in animal models. Deglycosylated Ig appeared to be unable to provide anti-inflammatory protections, although glycan was found in only 1–3% of IgG preparation (35). Sialylated Fc present in low amount in IVIg binds to SIGN-R1 on macrophages leading to an increased expression of the inhibitory FcγRIIB receptors and decreased expression of FcγRs. The overall result is an anti-inflammatory and anti-phagocytic effect. The key role of the terminal sialic acid residues is confirmed by the role of the molecules CD22 and CD33, also called SIGLEC, which are expressed by B-cells and cells of innate immune system (36, 37). Their capacity to trigger cell inactivation is related to the activation of intracellular immunoreceptor tyrosine-based inhibitor motifs (ITIMs). Of note, while the importance of Fc sialylation has been clearly shown in animal models, its role in humans should be better evaluated, and important species differences may probably exist.

## Conclusion

Immunoglobulin therapy is a consistently evolving practice that provides life-saving protection to patients with PID, particularly with antibody deficiency as well as in patients with other immune-mediated diseases. Taking into account the growing demand of Ig preparations not only for PID patients but also for subjects suffering from chronic immune-mediated diseases, more clinical trials will be required as well as basic research able to improve the knowledge on the mechanisms of action of IVIg.

## Conflict of Interest Statement

The authors declare that the research was conducted in the absence of any commercial or financial relationships that could be construed as a potential conflict of interest.
